# Sensor-Acquired Reachable Workspace (RWS) Correlates with Activities of Daily Living (ADL) Function in Stroke as Measured by Functional Independence Measure (FIM) Self-Care

**DOI:** 10.3390/s24216786

**Published:** 2024-10-22

**Authors:** Vicky Chan, Seungcheol Austin Lee, Jaylen M. Lee, Jay J. Han

**Affiliations:** 1 Department of Physical Medicine and Rehabilitation, University of California at Irvine School of Medicine, Irvine, CA 92617, USA; jayjhan@hs.uci.edu; 2 College of Media and Communication, Texas Tech University, Lubbock, TX 79409, USA; s.austin.lee@ttu.edu; 3 Biostatistics, Epidemiology & Research Design Unit at the Institute for Clinical and Translational Sciences (ICTS), University of California at Irvine, Irvine, CA 92697, USA; jaylenl@uci.edu

**Keywords:** stroke, reachable workspace, upper extremity, activities of daily living, function

## Abstract

Optimal upper extremity motor control and range of motion are necessary to achieve even the basic activities of daily living (ADL) function. Stroke, with resulting hemiparesis, can significantly and negatively impact an individual’s ADL function. Functional Independence Measure (FIM) self-care score can provide an assessment of what aspects and to what degree ADL functions are impaired. FIM self-care assessment can also track changes in ADL function during stroke recovery and rehabilitation. Recently, the sensor-acquired 3D motion analysis of stroke patients’ upper extremity has shown promise as a potential alternative to assess ADL function. This observational study evaluates whether the sensor-acquired upper extremity reachable workspace (RWS) measure correlates with clinician-evaluated FIM self-care score in stroke patients. Seventeen patients with stroke were enrolled in the study. FIM self-care, NeuroQoL upper extremity, and reachable workspace outcome measures (relative surface area, RSA) were collected upon rehabilitation hospital admission, at discharge, and at the 3-month visit. Pearson and Spearman’s rank correlation coefficients as well as multiple linear regression analyses were used to determine the relationships between FIM self-care, NeuroQoL, and reachable workspace RSAs. Moderately strong correlation between total reachable workspace RSA and total FIM self-care score at discharge and at 3 months were noted (r = 0.619, r = 0.661, *p* < 0.05), and similarly strong correlation was also noted with the upper extremity NeuroQoL total score (r = 0.690, r = 0.815, *p* < 0.05). Multiple linear regression analyses revealed a change in average bilateral total RSA of 0.1 unit from admission to the 3-month follow-up correlated with a respective change in the FIM self-care score of 2.011 points (95%CI: 0.663–3.360). Longitudinal improvement in ADL function during stroke rehabilitation and recovery process is correlated with improvement in reachable workspace.

## 1. Introduction

Stroke with resulting hemiplegia of upper and lower extremities can significantly impact an individual’s mobility and activities of daily living (ADL) functions. It is reported that the majority of stroke patients (~85%) suffer from upper extremity impairment affecting their ADLs [1]. Significant physical impairment and disability of upper extremity after stroke leads to loss of functional independence for the affected patient, decreased ability to perform basic self-care, and reduced quality of life [2]. For some patients with stroke after their acute medical hospitalization, additional comprehensive inpatient rehabilitation may be needed to facilitate recovery after stroke and optimize rehabilitation efforts [3]. Therefore, the accurate and clinically meaningful assessment of stroke patients’ upper extremity function and quantifying change over time are important in stroke rehabilitation.

The standard of care during rehabilitation includes tracking outcome measures such as the Functional Independence Measure (FIM) or other similar outcome measures upon admission and at discharge from the rehabilitation hospitalization. The FIM instrument was developed in 1983 and until recently has served as the mainstay standard functional outcome measure to evaluate patients with various physical impairments [4]. Recently, in the U.S., Quality Indicator (QI) has been adopted as the standard measure for post-acute-care rehabilitation [5]; however, the underlying conceptual basis for both FIM and QI remains similar in that both assess/grade the functional status of an individual based on the level of assistance that a person requires (based on clinician evaluation) [6]. Patient-reported outcome (PRO) measures can further shed light on patient’s own experience of functional impairment as well as self-perceived impact on daily functional activities. The NeuroQoL (Quality of Life in Neurological Disorders) questionnaire is one of several available self-reported assessments of functional impairment that can help quantify a patient’s perceived function as well as facilitate tracking any clinically meaningful changes [7].

With advances in health-related technologies, clinicians are increasingly exploring ways to leverage the use of these tools in health care applications. In the rehabilitation realm, unobtrusive, simple, and low-cost motion capture sensor systems (such as Kinect, Microsoft, Redmond, WA, USA) have been utilized to assess a patient’s upper extremity range of motion, and in conjunction with kinematic modeling and software programs, are able to reconstruct an individual’s three-dimensional (3D) upper extremity reachable workspace (RWS) [8].

At this time, the validity and reliability of the upper extremity RWS outcome measure (relative surface area, RSA) has been extensively investigated and demonstrated its clinical usefulness in various neuromusculoskeletal conditions including muscular dystrophies, neuropathies, orthopedic conditions, and in elderly populations (20+ research publications to date) [9,10,11]. The potential application and utility of the RWS outcome measure in the stroke population have also been recently demonstrated [12,13]. In a study by our colleagues with 41 stroke patients, the total RSA of the paretic side correlated well with the Fugl-Meyer Assessment Upper Extremity (FMA-UE; R^2^ = 0.68, *p* < 0.01), the Motricity Index for Upper Extremity (MI-UE; R^2^ = 0.65, *p* < 0.01), and the Disabilities of the Arm, Shoulder, and Hand questionnaire (QuickDASH; R^2^ = 0.42, *p* < 0.01) [12]. Furthermore, RSA demonstrated its potential as a surrogate marker to reliably track the recovery of UE mobility post stroke. Increases in total RSA were observed with higher Brunnström recovery stages—a clinical measure of the recovery of coordinated movement after stroke [12]. In another study of 58 hemi-paretic stroke patients, the RWS ratio demonstrated very high correlations with the FMA-UE total and the proximal scores (FMA-UE total: r = 0.81, *p* < 0.001; proximal: r = 0.89, *p* < 0.001) [13]. However, thus far, the RWS correlations with actual UE function in daily living, as measured by an ADL functional measure (clinician-reported outcome, ClinRO, FIM instrument) and a patient-reported outcome (PRO) measure such as NeuroQoL upper extremity, have not yet been examined.

In this paper, in order to further extend its practical application and characterize the clinical meaningfulness of the reachable workspace outcome measure as it relates to actual daily living functions, we focus our attention on correlations between reachable workspace RSA and the clinician-assessed upper extremity ADL measure (FIM, self-care) as well as the patient’s self-reported assessment of ADL function (NeuroQoL, upper extremity), in a cohort of stroke patients with varying degrees of hemiparesis undergoing a rehabilitation program. It is also important to determine whether a functional outcome measure is sensitive enough to detect clinically meaningful change over time. Therefore, this study also examines whether longitudinal changes in FIM self-care as observed in recovering stroke patients with upper extremity hemiparesis is similarly reflected by changes in reachable workspace RSA (from longitudinal data collected at admission, discharge, and at 3 months).

## 2. Materials and Methods

### 2.1. Overall Study Protocol

This longitudinal observation study followed the STROBE guidelines for research [14]. The study recruited participants aged ≥18 years, from August 2017 to October 2019, admitted to a university hospital acute rehabilitation unit. FIM self-care score, NeuroQoL upper extremity score, and reachable workspace RSA data (from both stroke-affected and unaffected arms) were collected at three time points: upon admission, at discharge, and at the 3-month post-stroke follow-up visit. The 3-month post-stroke follow-up was selected to optimize motor recovery gain and to capture this motor improvement [15]. The study protocol was approved by the Institutional Review Board (IRB) for conduct of ethical research, and written informed consent was obtained before the start of the study procedures.

### 2.2. Study Participants

A total of 22 potentially eligible participants were screened and 17 participants, who met all the inclusion criteria, with stroke as the primary admission diagnosis, were enrolled in the study. Only those participants who were able to understand and follow all study instructions were enrolled. Consecutive participants admitted to the rehabilitation unit during the study period were screened, and those meeting all inclusion criteria were enrolled; no formal power analysis was performed. Demographic, baseline anthropometric, relevant clinical information, and study outcome measures were collected at the time of admission to the rehabilitation unit. Subsequent study measures were collected for 15 patients at discharge (2 patients were unavailable for RSA data collection at the time of discharge), and at the 3-month follow-up, data from 13 patients were collected (2 patients were lost to follow-up). Measurement errors were minimized by using a single evaluator throughout the study.

### 2.3. Outcome Measures

#### 2.3.1. Functional Independence Measure (FIM Self-Care)

The FIM instrument is a valid and reliable tool to assess an individual’s ability to perform ADLs [16]. Typically, patient function is assessed by a clinician using FIM at the start and at the end of a rehabilitation episode of care. The inter-rater reliability of FIM has been established at an acceptable psychometric performance level (intra-class correlation coefficient, ICC, ranging from 0.86 to 0.88) [17]. The concurrent validity with the Barthel Index (ICC > 0.83) have shown strong construct validity between the Barthel Index and items on FIM that measure functional limitation [16]. There are six subsections in FIM: self-care, sphincter control, transfers, locomotion, communication, and social cognition. For the purposes of this study, focus will be on the self-care section of FIM as the more relevant upper extremity functional measurement. The FIM self-care section includes six elements of functional assessment: eating, grooming, upper body dressing, lower body dressing, bathing, and toileting. FIM score can range from 1 to 7, with 1 being categorized as requiring total assistance and 7 being complete independence [18]. Therefore, the range of total score available for the FIM self-care section would be 6–42, with a higher score indicating higher function.

#### 2.3.2. NeuroQoL (Upper Extremity Function)

NeuroQoL is a validated questionnaire-based PRO and a self-report of health-related quality of life in 17 domains for adults and 11 for children with various neurological disorders including stroke [19,20,21]. Specifically, the NeuroQoL upper extremity function domain questionnaire assesses fine motor and ADL function. The questionnaire comprises 20-item questions with scaled scores to evaluate the severity of upper extremity functional impairment to perform various ADLs involving manual and upper extremity reach-related functions [19]. The participant answers the questionnaire on how they would self-assess their performance regarding a given task: 1. Unable to do; 2. With much difficulty; 3. With some difficulty; 4. With a little difficulty; or 5. Without any difficulty. Each response is assigned a number ranging from 1 to 5 depending on the respective response to the question. These numbers determine the raw score. The range of total scores would be 20–100, with a higher score indicating higher function.

#### 2.3.3. Upper Extremity Reachable Workspace Protocol and Analysis

**Figure 1:**
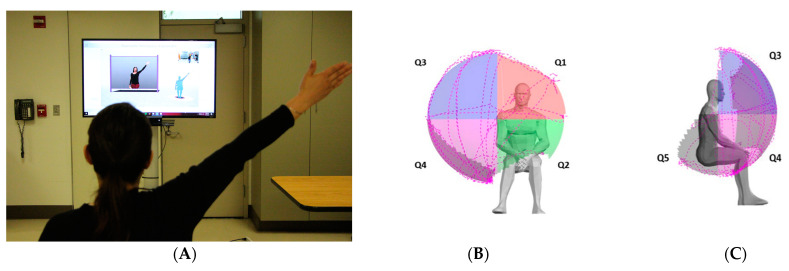
Reachable workspace with component quadrants. Reachable workspace system set up with a participant undergoing arm movement protocol in front of the video guide and Kinect sensor (**A**). An individual’s reachable workspace, reconstructed from the collected arm movement tracing illustrated by the dotted lines, and the visual output of relative surface area (RSA) envelope shown with four frontal quadrants Q1–Q4 (**B**) and one posterior inferior-lateral quadrant Q5 (**C**): Q1–Q4 are frontal quadrants viewed from the front (**B**); Q1, medial upper quadrant; Q2, medial lower quadrant; Q3, lateral upper quadrant; Q4, lateral lower quadrant; and Q5 lateral view (right side shown).

The upper extremity reachable workspace (RWS) measurement was performed using the Kinect 2.0 sensor following previously published protocols [8]. The method has demonstrated excellent reliability and validity across numerous studies in various patient populations by multiple different investigators [9,10,11]. Briefly, participants performed a set of standardized movements incorporating shoulder abduction, shoulder scaption, shoulder flexion, shoulder flexion across body, horizontal abduction, horizontal adduction, and shoulder extension. The second set of movements consisted of horizontal sweeps at the level of the umbilicus, shoulder, and above the head. These movements were designed to assess the reachability of outstretched arm to various locations in 3D space within each arm’s reach while the sensor tracked the arm movement, lasting about 1.5 min per arm (Figure 1). Each arm was tested separately, and the respective RWS measure was obtained. The arm motion was captured with a Kinect sensor located at a distance of 230 cm, with the participant seated in a standard upright chair without armrests. Each participant performed the standard arm movements while watching an instructional video that guided the participant through the movement protocol.

Following the previously published and established protocol for analysis [8,9,10,11], the frontal RWS envelope was split into four different quadrants with the shoulder joint serving as the origin: Q1 to Q4 and posterior inferior-lateral quadrant, designated as Q5. As previously described, to allow for comparison between patients, the absolute total and each quadrant’s reachable workspace surface envelope areas (m^2^) were normalized by each individual’s arm length to obtain the relative surface area, RSA [8]. The RSA results are displayed both numerically and visually with spatial mapping, with each frontal quadrant having a maximum value of 0.25 (the four frontal quadrants sum to 1.0) and with the addition of one posterior inferior-lateral quadrant contributing 0.25, resulting in a maximum value of 1.25 ‘for a total of five RSA quadrants’. For this study, the RSA values of each individual arm separately as well as an average of both arms from each study participant were used for analyses.

### 2.4. Statistical Analysis

The demographic and clinical characteristics of the study participants are presented as the mean and standard deviation for all continuous variables, and the dichotomous variable, such as sex, is presented as frequency and percentage. Pearson and Spearman’s rank correlation coefficients were used to determine the cross-sectional relationships between RSA and clinical outcome measures, including FIM self-care and NeuroQoL. Multiple linear regression was performed to investigate which quadrant, combination of quadrants, or total of all quadrants (Q1–Q5) is correlated with the change in clinical outcome measures of interest. Statistical analyses were conducted using SPSS version 29 (SPSS Inc., Chicago, IL, USA), with a *p*-value of <0.05 as the level of statistical significance. Additionally, all statistical significance was assessed through evaluating if the Benjamini–Hochberg adjusted *p*-values were less than 0.05 to correct for multiple comparisons [22]. Missing data was not included in the final data analysis.

## 3. Results

### 3.1. Study Participants Demographics

The baseline information of the participants and available clinical information are presented in Table 1. Seventeen participants at baseline demonstrated an average age of 62.76 years (SD = 12.46). Slightly less than half of the participants were male (41.2%, n = 7). Data from discharge and the 3-month follow-up showed an average age of 62.60 (SD = 12.96) and 65.23 (SD = 11.68) years, respectively. The initial mean National Institutes of Health Stroke Scale (NIHSS) was 9 for the available 15 of 17 participants, with a range of 2–20. The mean FIM self-care was 17.71 (SD = 5.57) at admission, which increased to 30.53 (SD = 7.03) at discharge and further increased to 37.15 (SD = 5.72) at the 3-month follow-up. The mean NeuroQoL upper extremity score was 69.06 (SD = 16.32) at admission, which increased to 76.73 (SD = 14.45) and further increased to 82.15 (SD = 14.91) at the 3-month follow-up.

### 3.2. Reachable Workspace (At Admission, Discharge, and 3 Months Post Stroke)

Reachable workspace data (RSAs) from each arm and the average of both arms were obtained for analyses. Extensive RSA data, comprising each quadrant (Q1–Q5) and the combined total RSA for each arm at admission, discharge, and at the 3-month follow-up, are available for review (Table 2).

**Figure 2:**
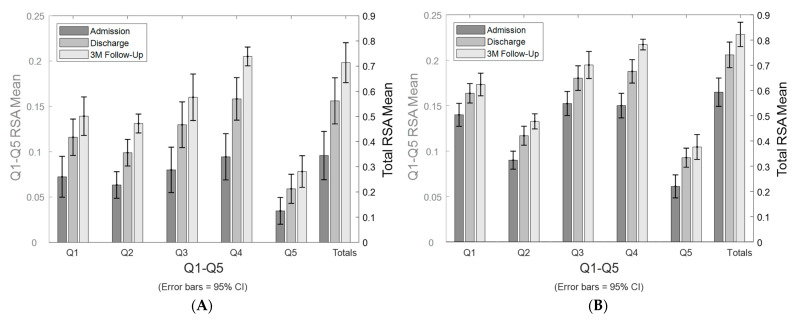
Stroke-affected paretic arm and bilateral arm reachable workspace change longitudinally. Changes in reachable workspace post stroke from admission, to discharge, to the 3-month follow-up. Bar graph of the mean RSAs from the stroke-affected arm, showing the individual quadrants’ RSAs (Q1–Q5) and total RSA (**A**). Bar graph of the mean RSAs of average bilateral arm data, showing the individual quadrants’ RSAs (Q1–Q5) and total RSA (**B**).

The study cohort’s mean RSAs of paretic and average bilateral arms by each quadrant and total RSA at admission, discharge, and 3 months are shown in Figure 2.

**Figure 3:**
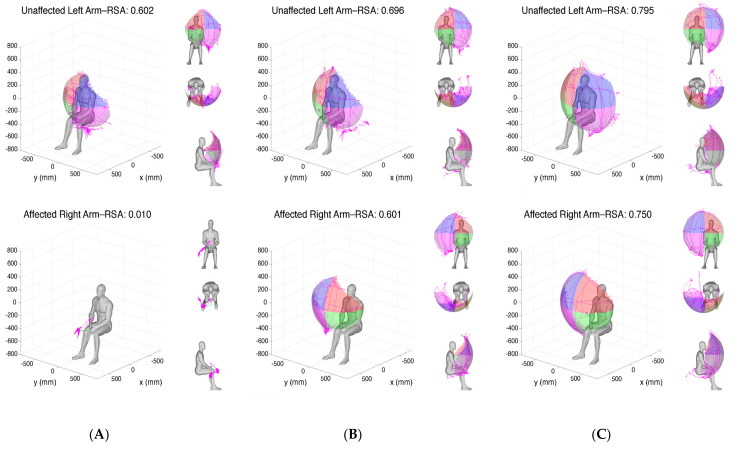
Reachable workspace upon admission, discharge, and 3 months post stroke. Graphical visualization of the bilateral relative surface area (RSA) of an example subject at admission (**A**), discharge (**B**), and at the 3-month follow-up (**C**) are shown. The top panels show the RSAs of the unaffected left arm and the bottom panels show the RSAs of the stroke-affected right side, gradually improving over time.

Overall, the RSAs of the unaffected arm remained stable throughout the course of the study, while the stroke-affected paretic arm’s RSAs showed significant initial reduction at admission with gradual improvement on discharge and at the 3-Month follow-up (example shown in Figure 3).

### 3.3. Reachable Workspace Measure (RSA) Correlation with FIM Self-Care and NeuroQoL

There is a moderate to fairly strong positive correlation between the individual quadrant RSAs and total RSA to FIM self-care score at admission (Table 3; Pearson correlation coefficient, r = 0.574 for Q1, r = 0.833 for Q2, r = 0.636 for Q3, r = 0.822 for Q4, r = 0.821 for Q5, and r = 0.812 for total RSA, *p* < 0.05). The correlation between total RSA and total FIM self-care score remains moderately strong at discharge and at the 3-month follow-up visit (r = 0.619 and r = 0.661, respectively, *p* < 0.05). For individual quadrant results, Q2, Q4, and Q5 show strong correlation with upper body dressing, lower body dressing, and toileting on admission, while Q1 and Q3 with less strong correlation. At discharge, both Q4 and Q5 show correlation with upper and lower body dressing. At the 3-month follow-up, Q2 shows correlation with bathing and toileting while Q5 correlates well with bathing and upper body dressing. There is also a fairly strong positive correlation between the total RSA and the NeuroQoL UE total score at discharge and 3 months (r = 0.690 and r = 0.815, respectively, *p* < 0.01).

### 3.4. Regression Analyses Evaluating Longitudinal RSA Change Compared to FIM Self-Care Change

Multiple linear regression was used to test if changes in RSA significantly correlated with FIM self-care changes over time. Evaluating changes from admission to discharge, regression analyses examining various RSA changes (each quadrant, total, upper, lower, medial, lateral, and other quadrant combinations) with FIM self-care changes (each ADL component and total) showed an overall positive relationship between the changes in bilateral RSAs compared to the changes in FIM self-care, but only Q4 and total FIM self-care score reached statistical significance (β = 8.045, *p* < 0.02).

**Figure 4:**
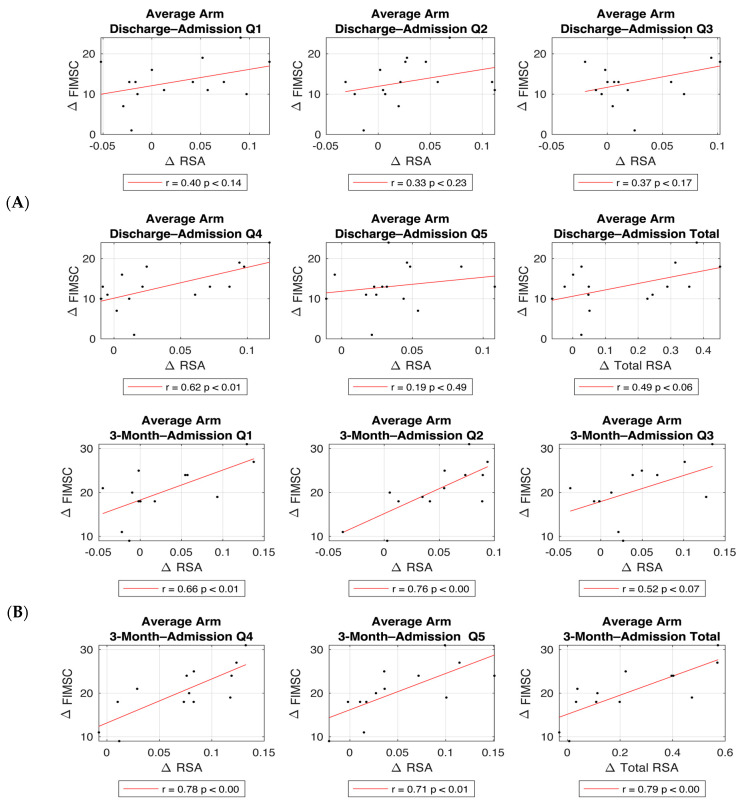
Correlation between RSA change and FIM self-care (FIMSC) change post stroke. Correlations between ∆RSA (Q1–Q5 and Total) and ∆FIMSC from admission to discharge (**A**), and from admission to the 3-month follow-up (**B**).

However, when looking at changes from admission to the 3-Month follow-up, significant correlations were found in all quadrants and total RSA changes (except Q3) with total FIM self-care change (Q1 r = 0.663, *p* < 0.013; Q2 r = 0.762, *p* < 0.002; Q3 r = 0.523, *p* < 0.067; Q4 r = 0.782, *p* < 0.002; Q5 r = 0.714, *p* < 0.006; total RSA r = 0.786, *p* < 0.001) (Table 4, Figure 4).

**Table 3:** Correlation between bilateral arm average RSA and FIM self-care and NeuroQoL at admission, discharge and at the 3-month follow-up.

FIM Self-Care at Admission (n = 17)	RSA Q1		RSA Q2		RSA Q3		RSA Q4		RSA Q5		Total RSA Q1–Q5	
	ρ	*p* value	ρ	*p* value	ρ	*p* value	ρ	*p* value	ρ	*p* value	ρ	*p* value
Eating	0.133	0.611	0.500	0.041 *	0.098	0.707	0.331	0.194	0.496	0.043 *	0.304	0.236
Grooming	0.740	0.777	0.226	0.384	0.050	0.848	0.281	0.274	0.318	0.213	0.196	0.450
Bathing	0.395	0.117	0.516	0.034 *	0.645	0.005 *†	0.740	<0.001 *†	0.538	0.026 *	0.650	0.005 *†
UB Dressing	0.535	0.027 *	0.813	<0.001 *†	0.452	0.068	0.859	<0.001 *†	0.858	<0.001 *†	0.782	<0.001 *†
LB Dressing	0.500	0.041 *	0.700	0.002 *†	0.542	0.025	0.854	<0.001 *†	0.611	0.009 *†	0.763	<0.001 *†
Toileting	0.508	0.037 *	0.716	0.001 *†	0.630	0.007 *†	0.737	<0.001 *†	0.678	0.003 *†	0.693	0.002 *†
	r	*p* value	r	*p* value	r	*p* value	r	*p* value	r	*p* value	r	*p* value
Total FIM Self-Care	0.574	0.016 *	0.833	<0.001 *†	0.636	0.006 *†	0.822	<0.001 *†	0.821	<0.001 *†	0.812	<0.001 *†
Total UE NeuroQoL	0.245	0.343	0.468	0.058	0.276	0.284	0.235	0.363	0.360	0.156	0.342	0.180
FIM Self-Care at Discharge (n = 15)	RSA Q1		RSA Q2		RSA Q3		RSA Q4		RSA Q5		Total RSA Q1–Q5	
	ρ	*p* value	ρ	*p* value	ρ	*p* value	ρ	*p* value	ρ	*p* value	ρ	*p* value
Eating	0.394	0.146	0.318	0.248	0.377	0.166	0.468	0.078	0.537	0.039 *	0.485	0.067
Grooming	0.529	0.043 *	0.523	0.045 *	0.512	0.051	0.668	0.006 *†	0.427	0.113	0.589	0.021 *
Bathing	0.399	0.140	0.619	0.014 *†	0.253	0.364	0.382	0.160	0.211	0.449	0.393	0.147
UB Dressing	0.585	0.022 *	0.572	0.026 *	0.572	0.026 *	0.659	0.008 *†	0.723	0.002 *†	0.721	0.002 *†
LB Dressing	0.601	0.018 *	0.577	0.024 *	0.573	0.026 *	0.700	0.004 *†	0.704	0.003 *†	0.737	0.002 *†
Toileting	0.334	0.223	0.561	0.030 *	0.202	0.470	0.504	0.055	0.370	0.175	0.457	0.087
	r	*p* value	r	*p* value	r	*p* value	r	*p* value	r	*p* value	r	*p* value
Total FIM Self-Care	0.595	0.019 *	0.677	0.006 *†	0.379	0.164	0.576	0.025 *	0.511	0.052	0.619	0.014 *†
Total UE NeuroQoL	0.582	0.023 *	0.820	<0.001 *†	0.384	0.158	0.618	0.014 *†	0.671	0.006 *†	0.690	0.004 *†
FIM Self-Care at 3-Month (n = 13)	RSA Q1		RSA Q2		RSA Q3		RSA Q4		RSA Q5		Total RSA Q1–Q5	
	ρ	*p* value	ρ	*p* value	ρ	*p* value	ρ	*p* value	ρ	*p* value	ρ	*p* value
Eating	0.759	0.003 *†	0.632	0.020 *	0.585	0.036 *	0.774	0.002 *†	0.774	0.002 *†	0.774	0.002 *†
Grooming	0.146	0.633	0.537	0.059	0.049	0.874	0.098	0.751	0.195	0.523	0.195	0.523
Bathing	0.491	0.089	0.668	0.012 *†	0.437	0.136	0.400	0.175	0.714	0.006 *†	0.641	0.018 *
UB Dressing	0.503	0.080	0.403	0.173	0.436	0.136	0.584	0.036 *	0.725	0.005 *†	0.617	0.025 *
LB Dressing	0.309	0.304	0.528	0.064	0.225	0.459	0.494	0.086	0.595	0.032 *	0.427	0.146
Toileting	0.167	0.586	0.748	0.003 *†	0.060	0.847	0.373	0.210	0.382	0.198	0.340	0.256
	r	*p* value	r	*p* value	r	*p* value	r	*p* value	r	*p* value	r	*p* value
Total FIM Self-Care	0.479	0.097	0.626	0.022 *	0.420	0.154	0.667	0.013 *†	0.771	0.002 *†	0.661	0.014 *†
Total UE NeuroQoL	0.668	0.013 *†	0.562	0.046 *	0.664	0.013 *†	0.867	<0.001 *†	0.828	<0.001 *†	0.815	<0.001 *†

ρ = Spearman’s rank correlation coefficient; r = Pearson correlation coefficient. * Statistically significant (* *p* < 0.05). † *p* values significant after Benjamini–Hochberg’s correction with False Discovery Rate at 5%. UB: upper body; LB: lower body; UE: upper extremity.

**Table 4:** Correlation between ∆bilateral arm average RSA and ∆FIM self-care assessment from admission to discharge and to the 3-Month follow-up.

∆ FIM Self-Care	∆RSA Q1		∆RSA Q2		∆RSA Q3		∆RSA Q4		∆RSA Q5		∆RSA Q1–Q5	
	r	*p* value	r	*p* value	r	*p* value	r	*p* value	r	*p* value	r	*p* value
Admission to Discharge	0.397	0.143	0.329	0.231	0.375	0.169	0.617	0.014 *†	0.195	0.486	0.492	0.063
Admission to 3-Month	0.663	0.013 *†	0.762	0.002 *†	0.523	0.067	0.782	0.002 *†	0.714	0.006 *†	0.786	0.001 *†

r = Pearson correlation coefficient. * Statistically significant (* *p* < 0.05). † *p* values significant after Benjamini–Hochberg’s correction with False Discovery Rate at 5%.

Additionally, multiple linear regression analyses examining various RSA changes (∆total, ∆each quadrant, and ∆quadrant combinations) from admission to the 3-month follow-up with FIM self-care changes (∆total and ∆each of the component ADLs) showed statistically significant correlations, as shown in detail in Table 5.

The most significant correlations were the following: ∆Total FIM Self-Care and ∆ Average of Two Arms Q1 β = 5.813, *p* < 0.046; ∆Total FIM Self-Care and ∆ Average of Two Arms Q2 β = 12.918, *p* < 0.014; ∆Total FIM Self-Care and ∆ Average of Two Arms Q4 β = 10.305, *p* < 0.01; ∆Total FIM Self-Care and ∆ Average of Two Arms Q5 β = 7.662, *p* < 0.006; ∆Total FIM Self-Care and ∆ Average of Two Arms Lateral Q3Q4Q5 β = 1.908, *p* < 0.012; and ∆Total FIM Self-Care and ∆ Average of Two Arms Total RSA Q1–Q5 β = 2.011, *p* < 0.008. The results of the analyses show that comparing two subpopulations, differing in change in total RSA (average of the sum of Q1 to Q5 bilaterally) from baseline to the 3-month follow-up by 0.1 units, who are otherwise similar with respect to baseline FIM self-care score, an estimated average difference in total FIM self-care score of 2.011 points is noted (95%CI: 0.663 points, 3.360 points, *p* < 0.008).

**Table 5:** Multiple linear regression analysis of ∆RSA of average bilateral arm correlation with ∆FIM self-care from admission to the 3-month follow-up.

∆RSA from Admission to 3-Month (n = 13)	∆Eating		∆Grooming		∆Bathing		∆UB Dressing		∆LB Dressing		∆Toileting		∆FIM SelfCare	
	β	*p* value	β	*p* value	β	*p* value	β	*p* value	β	*p* value	β	*p* value	β	*p* value
∆Bilateral Q1	0.705	0.097	−0.060	0.810	0.817	0.269	1.327	0.112	1.468	0.056	0.415	0.515	5.813	0.046 *
∆Bilateral Q2	0.920	0.174	−0.015	0.967	1.682	0.165	1.972	0.281	1.956	0.128	0.432	0.672	12.918	0.014 *†
∆Bilateral Q3	0.424	0.391	−0.092	0.729	0.821	0.319	1.126	0.175	1.532	0.063	0.563	0.399	4.918	0.118
∆Bilateral Q4	0.313	0.581	−0.026	0.932	1.919	0.066	1.974	0.097	2.465	0.014 *†	0.823	0.384	10.305	0.010 *†
∆Bilateral Q5	0.894	0.058	0.141	0.614	1.615	0.035 *	2.077	0.007 *†	1.822	0.021	0.690	0.300	7.662	0.006 *†
∆Bilateral Q1+3	0.306	0.194	−0.040	0.764	0.436	0.277	0.650	0.129	0.796	0.050	0.258	0.442	2.854	0.066
∆Bilateral Q2+4	0.290	0.564	0.055	0.832	0.348	0.672	0.361	0.674	0.653	0.457	0.110	0.874	2.238	0.495
∆Bilateral Q1+2	0.415	0.002 *†	−0.066	0.473	0.321	0.247	0.321	0.235	0.345	0.221	0.040	0.856	1.486	0.147
∆Bilateral Q3+4+5	0.300	0.010 *†	0.017	0.829	0.413	0.046 *	0.445	0.032 *	0.466	0.028 *	0.158	0.371	1.908	0.012 *†
∆Bilateral Total RSA Q1 to Q5	0.169	0.147	−0.004	0.954	0.328	0.099	0.497	0.031 *	0.470	0.017 *	0.159	0.362	2.011	0.008 *†

β = standardized beta coefficients. * Statistically significant (* *p* < 0.05). † *p* values significant after Benjamini–Hochberg’s correction with False Discovery Rate at 5%. UB: upper body; LB: lower body.

## 4. Discussion

With the sudden loss or serious impairment of hemi-body motor control resulting from a stroke, patients with stroke typically experience significant limitation in their ability to perform even the basic functions of daily activities. The impairment of upper extremity function, even on one side, can have a drastic impact on the performance of many ADL functions, some affected more than others. These coordinated movements of both upper extremities to varying degrees are often necessary for the efficient and successful performance of ADLs. Despite drastic initial negative impact on ADL functions soon after a stroke, with rehabilitation interventions and therapy, along with adaptive compensation strategies that occur in the context of natural stroke progression and recovery process, most patients with stroke regain some functions over time as seen by improvements in clinician-evaluated FIM scores. Similarly, this study’s stroke cohort’s FIM self-care score also improved over time, after inpatient stroke rehabilitation and at the 3-month follow-up.

Importantly however, the results of this study demonstrate for the first time that the change in stroke patients’ reachable workspace (RSA) correlates well with the observed change in FIM self-care score from admission to the 3-month follow-up. Specifically, in the study’s cohort of stroke patients after a rehabilitation program and undergoing functional recovery process, a 0.1 improvement in the average of total reachable workspace RSA bilaterally correlated to a 2.011-point improvement in FIM self-care score. Furthermore, the stroke cohort’s self-assessment of upper extremity function (and clinically meaningful ADL functional aspects to the patients themselves), as noted by NeuroQoL, correlated very well with reachable workspace RSAs. Together, this study’s findings suggest that the longitudinal tracking of reachable workspace RSA through an unobtrusive and relatively quick sensor-based evaluation is feasible in individuals with stroke and can provide clinically meaningful functional outcomes regarding real-life upper extremity ADL functions.

This is an important finding of this study since measuring and quantitatively tracking meaningful change in real-life ADL function has been challenging, as even the most basic daily living functional activities involve the complex interplay of component upper extremity motions at multiple joints working in a coordinated fashion to accomplish a given task. Until now, measuring an individual’s ability to perform multiple functional tasks has traditionally relied on clinical assessment by an experienced therapist or clinician to determine the FIM self-care score (or a similar tool such as QI), which takes into account complex movements that require both proximal and distal upper extremity range of motion, muscle strength, motor control, and dexterity. However, since adequate range of motion and motor control proximally at to the shoulder is necessary to locate the distal upper extremity in 3D space (within an individual’s reach) to accomplish various functional tasks, an upper extremity functional measure that incorporates both shoulder motion and reachability may serve as a viable surrogate marker for ADL function [8]. The importance of shoulder range of motion to achieve various daily-life functions has been reported previously. According to Safaee-Rad et al., the necessary shoulder complex motion for someone to eat includes shoulder flexion to 36 degrees, abduction to 22 degrees, medial rotation to 18 degrees, and horizontal adduction of 87 degrees [23]. Furthermore, according to Matsen et al., one must have a shoulder extension of 38 degrees and a horizontal abduction of 86 degrees in order to reach the perineum for hygiene tasks [24]. As shown in this study, longitudinal improvements in overall FIM self-care and NeuroQoL at the 3-month follow-up were closely reflected by improvements in RSA, providing added support for the validity and clinical meaningfulness of the reachable workspace outcome measure in stroke patients.

However, in stroke, it is important to keep in mind that even when one upper extremity is impaired, the unaffected upper extremity may be utilized to accomplish many of the ADL functions. Additionally, as stroke recovery progresses, due to the learned utilization of compensatory maneuvers in the paretic arm along with using the unaffected arm, an individual may be able to accomplish the necessary tasks albeit in an atypical way. Therefore, evaluating the combined reachable workspace of the bilateral upper extremity in addition to the impaired extremity would be important to correlate with actual ADL functions.

In a previous study evaluating the initial feasibility and clinical applicability of reachable workspace in a stroke population, only RSA in the stroke-affected paretic arm and its correlation to various upper extremity impairment measures were examined [12]. When comparing sensor-captured upper extremity motion measures such as RWS to functional assessments like FIM and NeuroQoL which incorporate many different bilateral ADL tasks, the global nature of bilateral upper extremity movement needs to be considered. When a stroke patient has limitations of self-care, compensation by the unaffected arm will instinctively come into play and will ultimately be used to affect the functional assessment score or self-reported measure of function. To account for this bilateral aspect of upper extremity function, combined RSAs from bilateral arms may be able to better estimate ADL function in stroke patients, and indeed this is supported by this study’s findings.

Additionally, the relative degree of functional impairment in the stroke-affected paretic distal upper extremity (hand dexterity or fine motor control) will significantly impact an individual’s ability to perform ADL tasks. Although the reachable workspace outcome measure does not directly assess the distal hand function, it nevertheless appears to serve as a surrogate measure that correlates relatively well with overall upper extremity function. This may be the case in general, as motor control and range of motion proximally at the shoulder improve post stroke, and the likelihood of improved distal hand function also increases through the typical stages of stroke recovery. Indeed, this correlation between RSA and distal hand function was also noted in a stroke population by Lee et al. in their study [12].

Another novel aspect of this study has been the incorporation of the posterior inferior-lateral quadrant, Q5, in the characterization of an individual’s functional reachable workspace. Up until now, frontal four quadrants, Q1–Q4, have been utilized to describe an individual’s reachable workspace. However, this study shows that incorporating this reachability into posterior inferior-lateral space (Q5) is important and allows for a fuller and more realistic characterization of the shoulder joint and distal arm movements that are necessary for dressing, reaching back, and toileting functional tasks. This study’s findings confirm that Q5 RSA is correlated with both the FIM self-care scores and the self-reported upper extremity function by NeuroQoL.

An interesting finding of this study is that NeuroQol at admission post stroke did not correlate well with RSA, but the correlation steadily improved and became stronger over time. By discharge and at the 3-month follow-up, essentially all reachable workspace quadrants and total RSA correlated extremely well with NeuroQoL (r = 0.815, *p* < 0.001). At this time, it is not completely clear as to why the initial admission NeuroQoL does not closely match RSA, given the severe degree of upper extremity impairment that a patient experiences right after the stroke and the accompanying severely limited reachable workspace. However, the authors surmise that a patient who undergoes such a sudden loss of previously normal functioning upper extremity (due to the sudden nature of stroke) may not be able to fully grasp the extent of functional limitations in the beginning stages of stroke, and therefore, a self-reported questionnaire outcome such as NeuroQoL obtained so early in the stroke process may not be representative of the actual functional impairment experienced by the patient.

The limitations of this study include the relatively small sample size; however, despite that, the primary study questions regarding reachable workspace RSA and its relationship to FIM self-care and NeuroQoL were able to be adequately addressed in this study. In the future, a study with a larger sample size and with a longer follow-up may be able to provide additional information through subgroup analyses of patients with differing degrees of stroke severity or in different stages of stroke recovery, and to further characterize the longitudinal sensitivity of RSA to ADL functional changes. Another limitation of this study may be the lack of a valid, reliable, and sensitive distal upper extremity outcome measure (a hand/manual dexterity measure which the outcome field lacks) that can complement the proximal upper extremity reachable workspace outcome measure to address the upper extremity functional assessment more fully and accurately. In the future, developing this combination of proximal and distal upper extremity functional outcome modules, which can combine to provide a more detailed characterization of the upper extremity function, may be a productive research direction. Additionally, the quality of upper extremity movement can be collected during the sensor-acquisition of arm motion; however, the effective incorporation of this information (such as tremor, ataxia, or spasticity) with reachable workspace has not yet been fully developed. Lastly, the utility of the FIM instrument to assess a patient’s function in clinical settings is declining as the newer Quality Indicator (QI) has become the standard measure in the U.S. Therefore, the applicability of this study’s findings correlating RSA with FIM self-care may not be as directly impactful. However, since both FIM and QI stem from similar underlying conceptual bases that grade the functional status of an individual based on the level of assistance that person requires for various ADLs, this study’s findings can likely be extended to QI. Further studies specifically looking at the correlation between RSA and QI may be needed to confirm this.

## 5. Conclusions

In conclusion, upper extremity ADL functional changes that occur after stroke and during stages of motor recovery can be determined by the reachable workspace outcome measure. Relatively quick to obtain and unobtrusive, the sensor-acquired upper extremity reachable workspace measure shows promise as a clinically meaningful and sensitive outcome measure, capable of evaluating ADL impairment as well as providing valuable information regarding the extent of functional disability experienced by individuals with stroke.

## Figures and Tables

**Table 1:** Demographic characteristics of the study participants (n = 17) at admission, (n = 15) at discharge, and (n = 13) at the 3-Month follow-up.

	Admission (n = 17)	Discharge (n = 15)	3-Month Follow-Up (n = 13)
Age, yrs. (mean ± SD)	62.76 ± 12.46	62.60 ± 12.96	65.23 ± 11.68
Age range, yrs. (min, max)	41, 84	41, 84	45, 84
Sex (n, %)	7 (41.2%) male	6 (40%) male	6 (46.2%) male
	10 (58.8%) female	9 (60%) female	7 (53.8%) female
Initial NIHSS, mean (min, max)	9 (2, 20) *		
Self-care FIM (mean ± SD)	17.71 ± 5.57	30.53 ± 7.03	37.15 ± 5.72
NeuroQoL (mean ± SD)	69.06 ± 16.32	76.73 ± 14.45	82.15 ± 14.91
LOS rehabilitation (mean ± SD)		18.87 ± 12.06	
Type of stroke, n Ischemic	13	12	10
Hemorrhagic	4	3	3
Hemiplegic side, n			
Right	7	6	5
Left	10	9	8

SD = Standard deviation. NIHSS = National Institutes of Health Stroke Scale. LOS = Length of stay for inpatient stroke rehabilitation. * 12 participants only. Missing data of five patients transferred from outside facility or without initial NIHSS.

**Table 2:** (**A**) The mean relative surface area (RSA) of the stroke-affected arm by individual and total quadrants at admission, discharge, and at the 3-Month follow-up. (**B**) The mean relative surface area (RSA) of the non-paretic arm by individual and total quadrants at admission, discharge, and at the 3-month follow-up.

**(A)**			
Affected Side RSA	Admission (n = 17)	Discharge (n = 15)	3 Month Follow-Up (n = 13)
	(Mean ± SD)	(Mean ± SD)	(Mean ± SD)
Quadrant 1	0.072 ± 0.090	0.116 ± 0.075	0.139 ± 0.073
Quadrant 2	0.063 ± 0.059	0.099 ± 0.055	0.131 ± 0.036
Quadrant 3	0.080 ± 0.100	0.130 ± 0.094	0.160 ± 0.089
Quadrant 4	0.094 ± 0.102	0.158 ± 0.088	0.205 ± 0.036
Quadrant 5	0.035 ± 0.059	0.059 ± 0.060	0.078 ± 0.060
Total (Q1–Q5)	0.345 ± 0.384	0.562 ± 0.343	0.714 ± 0.275
**(B)**			
Unaffected Side RSA	Admission (n = 17)	Discharge (n = 15)	3 Month Follow-Up (n = 13)
	(Mean ± SD)	(Mean ± SD)	(Mean ± SD)
Quadrant 1	0.207 ± 0.032	0.211 ± 0.033	0.207 ± 0.026
Quadrant 2	0.117 ± 0.037	0.135 ± 0.030	0.134 ± 0.042
Quadrant 3	0.225 ± 0.029	0.231 ± 0.027	0.229 ± 0.023
Quadrant 4	0.206 ± 0.024	0.217 ± 0.017	0.229 ± 0.011
Quadrant 5	0.087 ± 0.053	0.126 ± 0.039	0.131 ± 0.049
Total (Q1–Q5)	0.842 ± 0.124	0.920 ± 0.096	0.931 ± 0.103

## Data Availability

The data presented in this study are available on request from the corresponding author. The data are not publicly available due to privacy.

## List of Abbreviations

3D	Three-dimensional
ADL	Activities of daily living
ClinRO	Clinician-reported outcome
FIM	Functional Independence Measure
ICC	Intraclass Correlation Coefficient
IRB	Institutional Review Board
NeuroQoL	Quality of Life in Neurological Disorders
NIHSS	National Institutes of Health Stroke Scale
PRO	Patient-reported outcome
QI	Quality Indicator
RWS	Reachable workspace
ROM	Range of motion
RSA	Relative surface area
STROBE	Strengthening the Reporting of Observational Studies in Epidemiology
